# Association of Microcalcification Clusters with Short-term Invasive Breast Cancer Risk and Breast Cancer Risk Factors

**DOI:** 10.1038/s41598-019-51186-w

**Published:** 2019-10-10

**Authors:** Maya Alsheh Ali, Kamila Czene, Per Hall, Keith Humphreys

**Affiliations:** 1 Department of Medical Epidemiology and Biostatistics, Karolinska Institutet, Stockholm, Sweden; 2 Swedish eScience Research Centre (SeRC), Stockholm, Sweden

**Keywords:** Risk factors, Epidemiology

## Abstract

Using for-presentation and for-processing digital mammograms, the presence of microcalcifications has been shown to be associated with short-term risk of breast cancer. In a previous article we developed an algorithm for microcalcification cluster detection from for-presentation digital mammograms. Here, we focus on digitised mammograms and use a three-step algorithm. In total, 253 incident invasive breast cancer cases (with a negative mammogram between three months and two years before diagnosis, from which we measured microcalcifications) and 728 controls (also with prior mammograms) were included in a short-term risk study. After adjusting for potential confounding variables, we found evidence of an association between the number of microcalcification clusters and short-term (within 3–24 months) invasive breast cancer risk (per cluster OR = 1.30, 95% CI = (1.11, 1.53)). Using the 728 postmenopausal healthy controls, we also examined association of microcalcification clusters with reproductive factors and other established breast cancer risk factors. Age was positively associated with the presence of microcalcification clusters (p = 4 × 10^−04^). Of ten other risk factors that we studied, life time breastfeeding duration had the strongest evidence of association with the presence of microcalcifications (positively associated, unadjusted p = 0.001). Developing algorithms, such as ours, which can be applied on both digitised and digital mammograms (in particular for presentation images), is important because large epidemiological studies, for deriving markers of (clinical) risk prediction of breast cancer and prognosis, can be based on images from these different formats.

## Introduction

Microcalcifications are deposits of calcium oxalate and calcium phosphate within the breast tissue that appear as white specks on a mammogram. The mechanisms by which microcalcifications occur are not clearly understood, although many factors are suspected to play a role, such as age, hormonal unbalance, pregnancy, breastfeeding and diet change. Active cellular processes, or effects of cellular degeneration may be involved^1^. Calcification deposits are found within the ductal system, the breast acini, stroma and vessels^2^. Microcalcifications are present in approximately 55% of nonpalpable breast malignancies and are responsible for the detection of 85–95% of cases of ductal carcinoma *in situ* (DCIS) by screening mammography^3^, and they can also be present in invasive cancers^4^. The role of microcalcifications in the detection of breast cancer has been widely studied and some research groups have even investigated the role of microcalcifications in terms of risk and progression of breast cancer.

Previous studies have shown that women with false-positive results at screening have, on average, higher risks of breast cancer being detected at subsequent mammographic examinations than women with negative screening results^5–7^, in particular, when the false positive results are due to microcalcifications at mammography^8^. Detected abnormalities, although noncancerous, may therefore be useful as imaging features for breast cancer risk prediction. Microcalcifications represent a challenge in both perception and interpretation; between 12.7% and 41.2% of women are recalled with calcifications as the main mammographic feature/finding in screening programs. Although the value of using the computer aided detection (CAD) in breast screening is questioned^9^, in a study in the U.S., where CAD is used routinely in screening, it has been shown that false positive recall is associated with short-term risk of breast cancer^10^. It has also been demonstrated that CAD generated false positive detection, which includes the assessment of the presence of suspicious microcalcifications, can be used as a quantitative imaging marker of short-term breast cancer risk^11^. Further, it has been shown in a population where CAD is not routinely used, that the presence of clustered microcalcifications detected by a CAD algorithm, is important for short-term risk prediction^12^, even of invasive breast cancer.

Although almost all mammograms, nowadays, are acquired with full-field digital mammography systems (and are available in either for-processing or for-presentation formats, or both), some valuable databases contain predominantly traditional screen-film mammographic images. In the current paper, we adapt a method that we recently developed for detecting microcalcification clusters in for-presentation digital images^13^, for use on digitised images. Developing such algorithms as ours, which can be applied on both digitised and for-presentation digital mammograms, is important because large epidemiological studies, for deriving markers of (clinical) risk prediction of breast cancer and for studying prognosis, may have to be based on banks of images from different formats. We evaluate our algorithm in an epidemiological study of the short term risk of breast cancer using a case-control study design.

Our algorithms are specifically intended to be used in studies aimed at improving understanding of the importance/potential of microcalcifications for short-term risk prediction. Unlike in the development of CAD algorithms for suspicious microcalcification detection, in our context there is no gold-standard that can be derived and trained against. The underlying logic of our approach is that even if no detectable tumor is currently present, there may be characteristics in a woman’s mammographic image, picked up by our algorithm, that are indicative of an increased risk of breast cancer in the near future (the coming few years). It would of course be possible to use existing CAD algorithms, e.g. based on deep learning in short-term risk prediction models (see Mordang *et al*.^14^ for a deep learning approach to microcalcification detection, trained against radiologists annotations, in for-processing digital images, and see Wang & Yang^15^ for a deep learning approach to distinguish between benign and malignant calcifications), but they may not be optimal for our purpose. CAD algorithms are designed specifically for cancer detection and are focused on malignant calcifications. However, even non-malignant calcifications may be indicative of a future risk.

To shed some light on the processes generating the microcalcification clusters that are picked up by our algorithm and to further demonstrate the algorithm’s validity, we also study association with classical breast cancer risk factors.

In summary, the purposes of our study are (1) to describe the adaptation of our algorithm (described previously for for-presentation digital images) for potential microcalcification cluster assessment on digitised mammograms and to investigate the association of these clusters with the short-term risk of developing invasive breast cancer, and (2) to investigate the associations of established breast cancer (mainly reproductive history) risk factors with the presence of microcalcification clusters on mammograms.

## Methods

### Materials

Our analysis is based on CAHRES (Cancer and Hormone Replacement Study), a population-based post-menopausal breast cancer case-control study of Swedish residents born in Sweden and aged 50 to 74 years, between October 1993 and 31 March 1995. The CAHRES study base, from which we selected a subset of cases and controls, includes approximately 6000 women (approximately 3000 cases and 3000 age group matched controls). Details on data collection in CAHRES have been described elsewhere^16^. All cases had primary invasive breast cancer. CAHRES was able to collect (via screening and radiology units) mammograms for approximately 75% of its cases^17^. After making a number of exclusions (see Eriksson *et al*.^17^) e.g. for having implants in the breast, a measure of percent density was able to be calculated for 1747 cancer patients, on mammograms close to diagnosis (the median time before diagnosis for these mammograms was 50 days). Area-based percent density (PD) has been measured for these women using Cumulus^18^, a computer-assisted thresholding technique, using the mammogram contralateral to the tumor. We were able to trace negative screens prior to diagnosis for 1338 of these cancer patients, but only 662 had a negative screening within 3−24 months before diagnosis. Of these, 325 had mediolateral oblique (MLO) mammograms from both left and right breasts. In the current study we used mammograms from 253 of these cases for which complete data on age, BMI, hormone replacement therapy (HRT) status, parity, smoking status, diabetes status, and age at menopause were available. We used a similar procedure, to that used for the cancer patients (cases), for selecting controls from the original CAHRES study base, by searching for images within 3−24 months before questionnaire date, and found 728 women that had both left and right mammograms as well as complete data for the covariates. In controls, PD had been measured from a single mammogram, with side chosen at random. We note that we could use fewer cases (253) than controls (728) because we needed the exact diagnostic date for cases, which was sometimes missing.

For both cases and controls, body mass index (BMI) was recorded at entrance to the study, whereas age, in this study, was defined as age at mammography. Mammograms were digitised with an Array 2905 HD Laser Film Digitizer (Array Corporation, Tokyo, Japan), which cover a range of 0−4.7 optical density. The size of the images was 4770 × 3580 pixels with 0.05 mm per pixel. Informed consent was obtained from all individual participants included in the study, and the study had the approval of the ethics review board at Karolinska Institutet, Stockholm, Sweden.

Additional information (with some missing values) on breast feeding, life time breast feeding duration, and age at first birth for parous women was included in an additional analysis of association with case-control status and also in our analysis of association of microcalcifications with breast cancer risk factors.

### Microcalcification cluster detection

**Figure 1:**
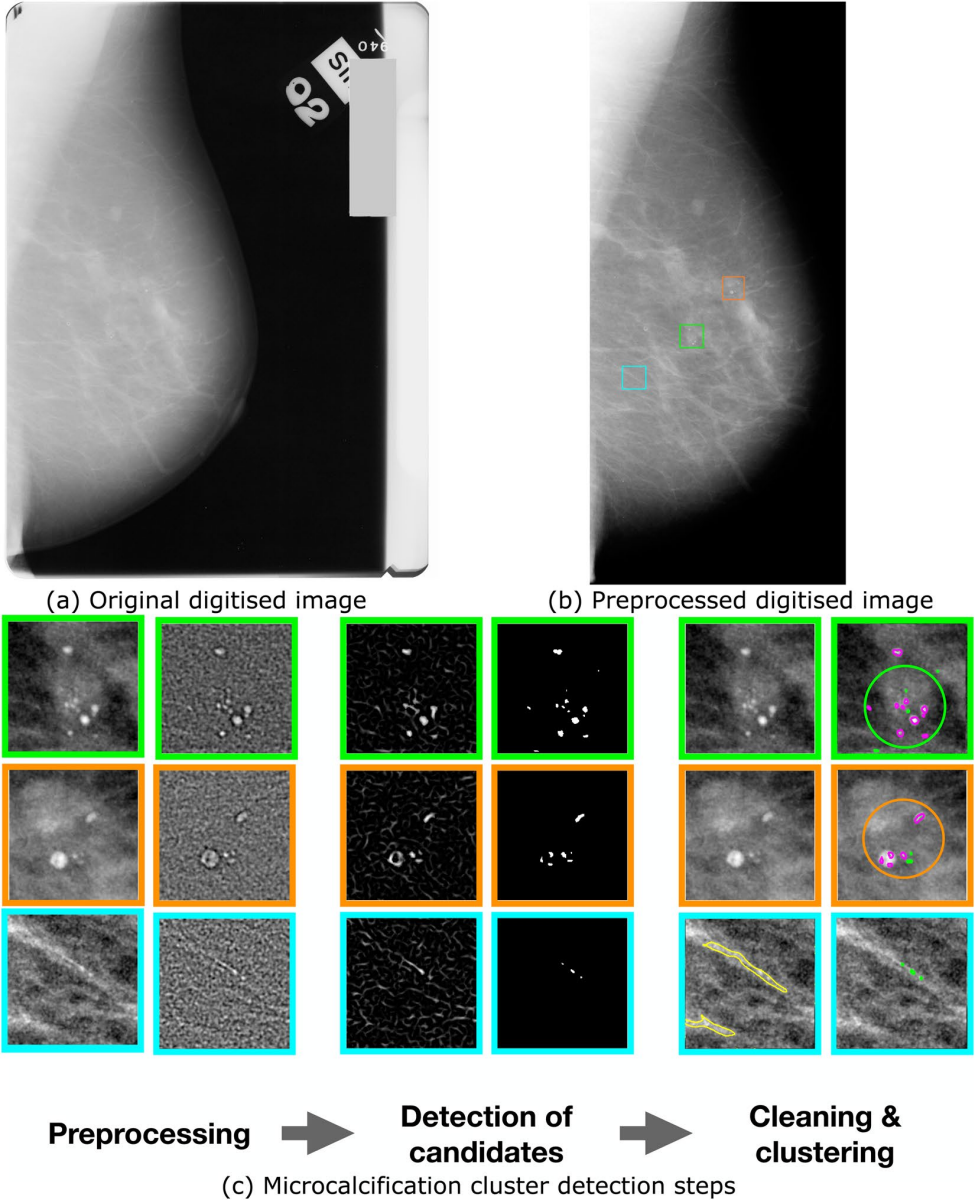
A schematic overview of the proposed method. (**a**) Shows the original digitised image before preprocessing and (**b**) shows the image after breast profile segmentation and denoising, (**c**) Shows the output of each step of microcalcification detection on three different patches. The preprocessing step includes the intensity transformation and the DOG filtering. The detection of candidates includes the HOG filtering and the thresholding. The final step includes removing noise and clustering of potential microcalcifications. Microcalcifications in magenta were retained whilst those in green were discarded in the cleaning step (clusters are in circles).

Our proposed approach for microcalcification cluster detection, which is described in details in our earlier paper^13^, can be divided into the following steps: (a) image preprocessing, consisting mainly of denoising, quality improvement and enhancement of small objects, (b) identification of microcalcifications, (c) filtering out noise and grouping microcalcifications into clusters; see Fig. 1. The main differences between digital for-presentation and traditional screen-film mammograms are in terms of image contrast, post-acquisition enhancement steps and the amount of noise. It is therefore mainly the preprocessing step of our algorithm for for-presentation digital images^13^ that needs to be adapted for use on screen-film mammograms.

#### Preprocessing of screen-film mammograms

The main aim of the preprocessing step is to separate the breast from other objects in the mammogram (i.e. labels, tags and screening artefacts, see Fig. 1(a)) with a minimum loss of breast tissue. To segment the breast profile, the contrast of the image first needed to be enhanced to brighten the low-intensity pixels close to the skin line. This was achieved using a logarithmic transformation of the image. The image was then segmented into three classes according to Otsu’s multithresholding method. The two brightest classes were kept since they corresponded to the breast region and the other objects in the mammogram, whilst the darkest corresponded to the background. The breast mask was finally extracted as the largest group of connected components and smoothed using morphological filtering.

In digitised mammograms, intrinsic noise can occur during the scanning, due to dust and artefacts present on the film or in the scanner. To reduce this noise, we fist applied morphological opening using a disk structuring element of one pixel radius and we then used the Anscombe transformation where noise was removed in the Anscombe domain through an adaptive Wiener filter^19^. From this step on, we used the same procedures as were used with for-presentation digital images^13^.

The microcalcifications are among the brightest objects in the image, so we spread out the upper gray levels (and contract the lower gray levels) by applying intensity transformations using the cosine function. We finally applied the Difference of Gaussian filter (DoG), a band-pass filter in which both cut-off frequencies were parametrised to be associated with microcalcification candidate sizes.

#### Microcalcification detection and grouping

The segmentation of microcalcifications in mammographic images is challenging due to several reasons. Due to their physical properties, they are arbitrarily shaped spots of a limited range of sizes and there are often strong variations in the background, which can introduce structures which are similar to those of microcalcifications. Also, a noisy background in an image can sometimes exceed the signal of interest. We tackled these problems by applying a selective detector (Hessian of Gaussian (HOG)), targeting both blob-like and line-like structures^20^, that can enhance the brightness of microcalcifications and, at the same time, give more homogeneous linear structures. After applying thresholding operations to obtain the microcalcification candidate, all objects with shapes, sizes and appearances similar to microcalcifications were then filtered to reduce the noise. The last step involved microcalcification grouping using a Density-Based Spatial Clustering of Applications with Noise (DBSCAN) algorithm^21^. The same parameters that were defined for for-presentation images^13^ were used here.

### Statistical analysis

We evaluated the association between the presence of microcalcification clusters and case-control status by fitting a logistic regression model treating case-control status as the dependent variable. We treated the total number of microcalcification clusters in the left and right breasts as an independent variable. The variable used in our study of for-presentation images was defined in the same way. We first fitted logistic regression using all cases (253) and controls (728) by including the covariates age (continuous), BMI (continuous), PD (continuous), HRT (categorical), parity (continuous), smoking status (categorical), diabetes status (categorical) and age at menopause (continuous), as adjustment variables. In an additional logistic regression analysis we also added breastfeeding and age at first birth as adjustment variables. Because these variables are defined only for non-parous women, we combined the variables parity, breastfeeding and age at first birth into a single categorical variable with 9 categories. We also calculated and compared AUCs for two models, with and without the total number of microcalcification clusters variable entered as a covariate. In both cases all other covariates, except breastfeeding and age at first birth were included (i.e. we used the first model). To avoid over-fitting, we calculated “honest” values (of the AUCs) using the bootstrap procedure described by Harrell *et al*.^22^. We note that the AUCs will be partially age-adjusted^23^ (i.e. will partly remove the effect of age), due to the fact that the study base, CAHRES, matched cases and controls within five year age groups.

To assess, at a population level, the association between a selection of established breast cancer risk factors (one at a time) with the presence of microcalcification clusters, using only breast cancer-free controls, we fitted a number of age-adjusted logistic regression models. We chose to study microcalcification clusters as a binary variable here (0 vs. 1+ microcalcification clusters). The data was too sparse to use multinomial logistic regression. Use of logistic regression is also consistent with a previous study of factors of vascular calcifications^24^. The risk factors were age, BMI, PD, parity, age at first birth, breastfeeding duration, breastfeeding, HRT use, smoking status, diabetes status and age at menopause, and these were treated in the same way as in the risk analysis. In the results section we report parameter estimates from the models as odds ratios (OR) (with 95% confidence intervals (CI)). All analyses were performed in accordance with relevant institutional and national guidelines and regulations.

### Consent for publication

All authors approved the manuscript and consented for publication.

## Results

We illustrate the results of applying our algorithm for detecting microcalcifications, for an example of digitised image; see Fig. 1. First, we segment the breast profile to remove tags and background noise. We denoise the image to improve its quality and we enhance the contrast of small objects. We show the output of each step on three different patches in Fig. 1(c): a group of microcalcifications in the first two rows, and a false detection caused by linear structures in the third row. After the identification of microcalcifications, we filter out noise and group microcalcifications into clusters since isolated calcifications are not clinically significant.

Key characteristics of cases and controls included in our association analyses are described in Table 1. There was strong evidence that both PD and HRT use are (positively) associated with case-control status.

**Table 1:** Key characteristics of individuals included in the case-control study of short term breast cancer risk.

Characteristic	Cases	Controls	P-value
Number	253	728	
HRT use			1 × 10^−08^
Never	152 (60.1%)	572 (78.6%)	
Past	6 (2.4%)	23 (3.1%)	
Current	95 (37.5%)	133 (18.3%)	
Age	62.277 (±6.682)	63.777 (±6.082)	0.016
BMI	25.855 (±3.819)	25.514 (±3.871)	0.225
PD	19.436 (±14.616)	14.612 (±13.696)	4 × 10^−06^
Smoking			0.009
Never	127 (50.2%)	434 (59.6%)	
Ever	126 (49.8%)	294 (40.4%)	
Parity	1.889 (±1.170)	2.163 (±1.444)	0.007
Age at first birth*	24.778 (±4.826)	24.927 (±4.743)	0.689
Breastfeeding*			0.227
Never	7 (3.8%)	37 (6.1%)	
Ever	179 (96.2%)	537 (93.9%)	
Breastfeeding duration*	10.234 (±6.750)	11.663 (±10.341)	0.089
Diabetes			0.539
No	237 (93.7%)	691 (94.7%)	
Yes	16 (6.3%)	37 (5.3%)	
Age at menopause	50.209 (±4.029)	50.191 (±4.057)	0.950

Means (with standard deviations) are given for continuous variables and proportions (with percentages) are given for categorical variables. P-values are obtained using likelihood ratio tests based on fitting logistic regression models without adjustment for additional covariates. ^*^Parous women only.

After adjusting for potential confounding variables (BMI, PD, HRT, parity, smoking status, diabetes status and age at menopause), we found evidence of an association between the total number of microcalcification clusters and short-term invasive breast cancer risk (per cluster OR = 1.306, 95% CI = (1.112–1.534), p = 1 × 10^−03^), see Table 2. The association for PD was OR = 1.022, 95% CI = (1.012–1.034), p = 1 × 10^−04^; this corresponds, for example, to an OR of 5.1 when comparing women with a difference in PD of 75%. In a second logistic regression where we adjusted for all potential confounding variables (210 cases and 605 controls), we found similar evidence of association with the number of microcalcification clusters (OR = 1.278, 95% CI = (1.069–1.529), p = 7 × 10^−03^). In this model the OR for PD was 1.025, 95% CI = (1.012–1.038), p = 1 × 10^−04^). Using a bootstrapping procedure (based on 1000 bootstrap samples), we obtained honest estimates of 0.656 and 0.645 for the AUCs for the full model, with and without the total number of potential microcalcification clusters, respectively (the corresponding apparent AUCs were 0.680 and 0.667).

**Table 2:** Analysis of association with short term breast cancer risk. Results from fitting a logistic regression model with case-control status as the dependant variable and BMI, PD, Parity and AFB, HRT and the number of potential microcalcification groups (*MCC*), coded as a continuous covariate.

Covariate	OR	(95% CI)	P-value
Age	0.982	(0.957, 1.007)	0.165
BMI	1.069	(1.026, 1.113)	2 × 10^−03^
PD	1.022	(1.012, 1.034)	1 × 10^−04^
Parity	0.859	(0.761, 0.969)	0.0135
HRT use			
Never		reference	
Past	0.607	(0.250, 1.607)	0.315
Current	2.274	(1.615, 3.203)	2 × 10^−06^
Smoking	1.425	(1.041, 1.952)	0.027
Diabetes	1.227	(0.633, 2.379)	0.545
Age at menopause	1.007	(0.969, 1.046)	0.724
MCC	1.306	(1.112, 1.534)	1 × 10^−03^

Among the 728 postmenopausal healthy women, 27% had microcalcification clusters at mammography. Age was positively associated with the presence of microcalcification clusters (Table 3). Results from the association analysis of the other risk factors with microcalcification clusters, based on fitting age-adjusted models are presented in Table 3. Analyses were carried out on subsets of individuals with complete data on the risk factors (one-at-a-time). Life time duration of breastfeeding was positively and significantly associated with the presence of microcalcification clusters (unadjusted p-value = 0.001). We found a suggestion of positive association with diabetes status (unadjusted p-value = 0.019) and of negative associations with smoking status, and age at first birth (unadjusted p-values of 0.006 and 0.044, respectively), but none of these associations withstood the (admittedly conservative) Bonferroni correction for multiple testing. No significant association was found between HRT use, PD, BMI, or age at menopause and the presence of microcalcification clusters.

**Table 3:** Results of tests of association between the existence of microcalcification groups and breast cancer risk factors, based on fitting logistic regression models with the existence of microcalcificaions treated as a dependant variable. Covariates are included one-at-a-time, along with age.

Covariate	OR	(95%CI)	P-value	*N*
Age	1.060	(1.026, 1.095)	4 × 10^−04^	728
BMI	1.002	(0.994, 1.011)	0.549	728
PD	1.001	(0.999, 1.004)	0.303	728
Parity	1.018	(0.996, 1.041)	0.106	728
Age at first birth	0.961	(0.924, 0.999)	0.046	647
Breastfeeding duration	1.031	(1.012, 1.051)	0.001	526
Breastfeeding	5.746	(1.356, 24.344)	0.018	572
HRT use				728
Never		reference		
Past	1.026	(0.853, 1.234)	0.780	
Current	1.043	(0.958, 1.135)	0.332	
Smoking	0.910	(0.852, 0.973)	0.006	728
Diabetes	1.194	(1.030, 1.383)	0.019	728
Age at menopause	1.004	(0.996, 1.012)	0.269	728

## Discussion

We have adapted an algorithm for detecting microcalcifications which was originally developed for for-presentation digital images, for use on digitised film mammograms and, in a case-control study of postmenopausal invasive breast cancer, using negative screening images taken prior to diagnosis, have shown that our measure of microcalcification clusters is significantly associated with short-term risk of invasive breast cancer. In this study, we provide further evidence that microcalcifications can predict the future risk of invasive breast cancer.

Many factors that increase the long-term risk of breast cancer are now known, such as a high percent density, a family history of breast cancer, a late age of first birth, an early onset of first menstruation, a late age at menopause. A large number of genetic markers of susceptibility to the disease, both rare and common have also been identified^25^. Whilst factors such as those listed above may be useful for long-term individualised screening^26^, there is also a need to find factors associated with short-term breast cancer risk, for which there would be an immediate clinical relevance (recalling women to screening earlier if they have a high short-term risk). As we, and others, have shown, incorporating potential microcalcification clusters on mammography can be helpful for short-term breast cancer risk prediction.

The association of the presence of microcalcification clusters and age can be due to involution^27^ or to noncancerous lumps shrinking and calcifying with menopause and aging. To our knowledge, ours is the first study to describe an association between breast feeding and the presence of microcalcification clusters. Several cases of postlactational microcalcifications have been reported in the literature^28,29^. This might be a result of a combination of programmed cell death (apoptosis) and stasis of secretions^29^, since upon cessation of lactation the breast decreases in size as the glandular elements regress and atrophy.

The mechanisms by which these factors are associated with microcalcifications is still not clear and it is possible that these factors are associated differently across subtypes of microcalcification clusters.

**Figure 2:**
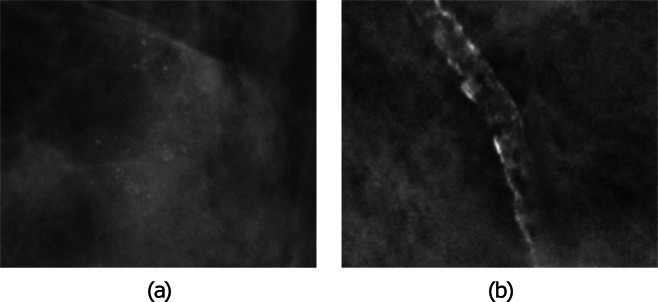
Examples of calcification: (**a**) shows microcalcifications, and (**b**) shows breast arterial calcifications.

In the current study, we considered all type of microcalcifications. For example, the output of our algorithm will include breast arterial calcifications (BAC), which are small calcium deposits in the vessel walls of the arteries; see Fig. 2(b). These are in fact one of the most common false positives detected by CAD systems. We note that these calcifications are a risk factor for cardiovascular disease and that studies have shown that increasing age, diabetes, parity and earlier age at first birth are associated with higher BAC prevalence^24,30^, whereas smoking is associated with lower BAC prevalence^30^.

The confidence intervals for the effect size of microcalcification clusters in this study (OR = 1.306; 95% CI: (1.112,1.534)) and in our previous study^13^ (OR = 1.510; 95% CI: (1.317,1.733)), which was based on for-presentation mammograms, largely overlap. We suspect, however, that the “true” effect size based on digitised images may be smaller due to the quality of the images, since digital mammograms offer improved image contrast, and no digitisation noise, as well as access to information about the exact pixel resolution of the images. We would also point out that the women considered in this study are older than in our previous study, which means that there is a higher rate of detected microcalcification clusters due to age. There are limitations to any detection process, regardless of image format; it is not possible to account for random artifacts and noise that occur during the acquisition/digitisation step. In addition, studies have shown that microcalcification detection is sensitive to image quality and the radiation doses that are used^31,32^.

We reiterate that it is important to develop algorithms for novel image-based markers that can be derived from both digital and digitised mammograms in order to open the door for research based on large collections of mammograms. We are, for example, currently working on a study of mammographic features for the early prediction of invasive breast cancer subtypes which is based on combining digital and digitised mammograms. Another interesting line of research would be to investigate the association of features describing microcalcification clusters, such as cluster locations in the breast, morphology and density, with cancer risk. It would even be interesting to study the relationships of these features with cancer risk factors. In a future study, we would like to test for association between microcalcification clusters and breastfeeding duration based on digital images.

## Conclusion

We have presented a method for microcalcification detection using digitised images. Our results confirm the association between the number of microcalcification clusters and the short-term risk of being diagnosed with invasive breast cancer. We have also reported associations between the presence of microcalcification clusters and a selection of breast cancer risk factors. Lifetime breast feeding duration was found to be positively associated with the presence of microcalcifications.
